# *ALPK3* gene mutation in a patient with congenital cardiomyopathy and dysmorphic features

**DOI:** 10.1101/mcs.a001859

**Published:** 2017-09

**Authors:** Ahmet Okay Çağlayan, Rabia Gonul Sezer, Hande Kaymakçalan, Ege Ulgen, Taner Yavuz, Jacob F. Baranoski, Abdulkadir Bozaykut, Akdes Serin Harmanci, Yalim Yalcin, Mark W. Youngblood, Katsuhito Yasuno, Kaya Bilgüvar, Murat Gunel

**Affiliations:** 1 Department of Medical Genetics, School of Medicine, Istanbul Bilim University, Istanbul 34394, Turkey;; 2 Departments of Neurosurgery, Neurobiology and Genetics, Yale School of Medicine, New Haven, Connecticut 06510, USA;; 3 Department of Pediatrics, University of Health Sciences, Zeynep Kamil Maternity and Childrens' Diseases Training and Research Hospital, Istanbul 34668, Turkey;; 4 Department of Pediatrics, School of Medicine, Istanbul Bilim University, Istanbul 34394, Turkey;; 5 Division of Pediatric Cardiology, Department of Pediatrics, Zeynep Kamil Maternity and Childrens’ Diseases Training and Research Hospital, Istanbul 34668, Turkey;; 6 Division of Pediatric Cardiology, Department of Pediatrics, School of Medicine, Istanbul Bilim University, Istanbul 34394, Turkey;; 7 Department of Genetics, Yale Center for Genome Analysis, Yale School of Medicine, New Haven, Connecticut 06510, USA

**Keywords:** hypertrophic cardiomyopathy

## Abstract

Primary cardiomyopathy is one of the most common inherited cardiac diseases and harbors significant phenotypic and genetic heterogeneity. Because of this, genetic testing has become standard in treatment of this disease group. Indeed, in recent years, next-generation DNA sequencing has found broad applications in medicine, both as a routine diagnostic tool for genetic disorders and as a high-throughput discovery tool for identifying novel disease-causing genes. We describe a male infant with primary dilated cardiomyopathy who was diagnosed using intrauterine echocardiography and found to progress to hypertrophic cardiomyopathy after birth. This proband was born to a nonconsanguineous family with a past history of a male fetus that died because of cardiac abnormalities at 30 wk of gestation. Using whole-exome sequencing, a novel homozygous frameshift mutation (c.2018delC; p.Gln675SerfsX30) in *ALPK3* was identified and confirmed with Sanger sequencing. Heterozygous family members were normal with echocardiographic examination. To date, only two studies have reported homozygous pathogenic variants of *ALPK3,* with a total of seven affected individuals with cardiomyopathy from four unrelated consanguineous families. We include a discussion of the patient's phenotypic features and a review of relevant literature findings.

## INTRODUCTION

Cardiomyopathy (CMP) is classified into primary and secondary forms based on the involvement of other organ systems (Maron et al. 2006). There are several different clinical subtypes of primary cardiomyopathy, with hypertrophic cardiomyopathy (HCM) and dilated cardiomyopathy (DCM) being the two most prevalent (Towbin and Bowles 2002; Ahmad et al. 2005). The annual incidence of pediatric cardiomyopathy is around 1.13 cases in 100,000 children younger than 18 yr of age and 8.34 cases per 100,000 infants (Wilkinson et al. 2011; Mestroni et al. 2016). HCM is characterized by left ventricular hypertrophy (LVH) and results in a range of cardiac output and hemodynamic abnormalities. HCM affects approximately 1 in 500 adults globally and is considered to be the result of mutations affecting the sarcomere proteins that comprise the heart's contractile apparatus (Maron et al. 1995; Elliott et al. 2008; Harvey and Leinwand 2011; Semsarian et al. 2015). Histological features of HCM include interstitial fibrosis and myocyte enlargement and disarray. DCM, on the other hand, is characterized by increased left ventricular end diastolic diameter (Elliott et al. 2008). Idiopathic DCM carries a prevalence likely exceeding 1 in 250 (Hershberger et al. 2013) and is histologically characterized by cardiomyocyte hypertrophy, loss of myofibrils, and interstitial fibrosis (Cahill et al. 2013). Both HCM and DCM are responsible for heart failure, arrhythmias, and sudden cardiac death at any age worldwide (Maron and Maron 2013); and DCM is the most common indication for cardiac transplantation (Stehlik et al. 2012). Furthermore, both HCM and DCM display locus and allelic heterogeneity, and most mutations lead to an autosomal dominant pattern of inheritance with variable expression and incomplete penetrance (Hershberger and Siegfried 2011; Yingchoncharoen and Tang 2014). However, other inheritance patterns have been reported, including X-linked (Towbin et al. 1993), mitochondrial (Zaragoza et al. 2011), and autosomal recessive transmission in DCM and X-linked and recessive in HCM (Burke et al. 2016).

Next-generation sequencing (NGS) technologies are being utilized to identify novel disease-causing genes to further elucidate the unsolved causes of cardiomyopathy (Ware et al. 2012). Recently, homozygous null variants in *ALPK3* were reported as a novel cause of severe pediatric cardiomyopathy in five patients from three unrelated consanguineous families by Almomani et al. (2016). A report by Phelan et al. (2016) described an additional two affected individuals with CMP and *ALPK3* mutation and demonstrated abnormal calcium transport in *ALPK3-*mutant cardiomyocytes. Furthermore, two individuals with heterozygous null variants in *ALPK3* from Almomani's report were diagnosed with an atypical form of HCM at a young adult age. Here we report a null variant in the autosomal recessively inherited cardiomyopathy gene *ALPK3,* which was identified in a patient who initially presented with DCM that later progressed to HCM.

## RESULTS

### Clinical Presentation and Family History

**Figure 1.**
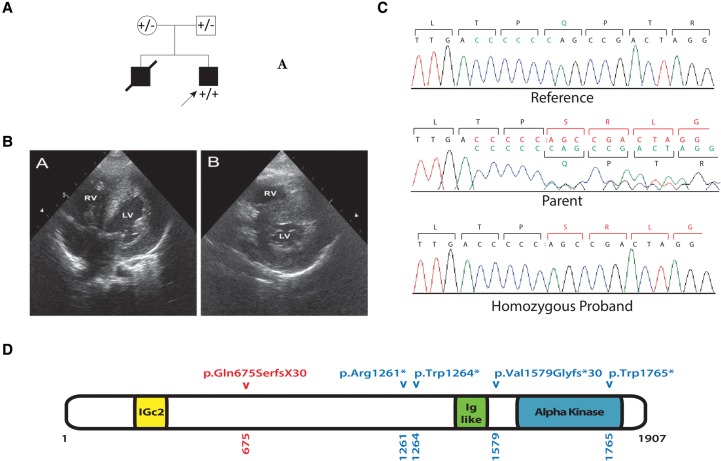
(*A*) Pedigree of the family demonstrating the affected siblings and, when available, their *ALPK3* genotypes. (*B*) Transthoracic echocardiogram of apical four-chamber view (*left*) showing symmetrically thickened left ventricular wall and ventricular septum. Parasternal short-axis view (*right*) showed diffuse concentric left ventricular hypertrophy. RV, right ventricle; LV, left ventricle. (*C*) Sanger sequencing results. Chromatographs obtained via Sanger sequencing analysis of the index patient and his parents. Sanger sequencing of wild-type control DNA was also performed. Note that the mutation identified via whole-exome sequencing was confirmed as being homozygous versus heterozygous, in the index patient versus his parents, respectively. The bases outlined in red in the wild-type sequence indicate the mutated base pairs. (*D*) A schematic representation of ALPK3, showing the functional and conserved domains of *ALPK3*. The red arrowhead indicates the location of the identified variant in this study. Blue arrowheads indicate previously reported variants.

The index case is a 2½-year-old male who was born preterm at 32 wk of gestation as a product of a reported nonconsanguineous union (however, parents were found to be from the same village with a small population during clinical evaluation of the patient). Family history was notable for a previous pregnancy in which the male fetus died because of cardiac abnormalities at 30 wk of gestation (Fig. 1A). However, no patient material from this previous fetus was available for clinical and/or laboratory examination. Fetal echocardiography at 21 wk of gestation of the index case demonstrated cardiomegaly (heart/thorax ratio of 0.8), decreased cardiac contractility, and thickened trabecular layer and moderator band. His end-diastolic interventricular septum diameter was measured at 3.9 mm, left ventricular end-diastolic diameter at 10.6 mm, and ejection fraction at 34%; a mild mitral valve insufficiency was also noted. A diagnosis of DCM was made. When he was 4 mo old, his weight was 5010 g (10th percentile), height was 59 cm (25–50th percentile), and head circumference was 39 cm (10th percentile). His examination revealed dysmorphic face including low-set ears and high arched palate. At the same age, two-dimensional echocardiography demonstrated diffuse left ventricular (LV) hypertrophy, with normal ejection fraction (LV end-diastolic diameter of 18 mm, end-systolic interventricular septum diameter of 7 mm, end-diastolic interventricular septum diameter of 5 mm, end-systolic LV posterior wall diameter of 9 mm, and end-diastolic LV posterior wall diameter of 6 mm). An LV outflow tract obstruction was not present on Doppler echocardiographic examination (Fig. 1B). These clinical findings were sufficient for a diagnosis of HCM. The parents were subsequently screened for cardiomyopathy with normal echocardiogram results.

### Genomic Analyses

#### Whole-Exome Sequencing

We performed whole-exome capture and sequencing of germline DNA obtained from the index case (Supplemental Table 1). Both parents were Sanger sequenced for the identified variants to complete segregation analysis. Whole-exome sequencing data were analyzed using an in-house bioinformatics pipeline as described in detail previously (Clark et al. 2013). We identified all exomic variants, classifying them as either single-nucleotide variants (synonymous, loss-of-function, or missense variants) or insertion/deletions, and we calculated both the heterozygous and homozygous population allele frequencies of each of the variants. We used a population cutoff of 0.01% to filter for candidate causative genomic events, after annotating each variant with its frequency in a Yale in-house exome sequence database (*n* > 5000) and the Exome Aggregation Consortium (ExAC), ESP6500 and the 1000 Genome databases. Using this approach, eight homozygous novel variants were identified in the homozygous-by-descent (HBD) genomic segments of the index case (Table 1). All but one (*TNK2*) of the variants that were identified in the affected individual segregated with the phenotype within the family. Notable variants included frameshift mutations in *ALPK3* and *KANSL1L* and a missense variant in the previously established X-linked deafness gene *POU3F*. The mutation in *ALPK3* was a homozygous 1-bp deletion (ENST00000258888.5: c.2018delC), resulting in a predicted premature termination (ENSP00000258888.5:p.Gln675SerfsX30) (Fig. 1C). This mutation segregated in the family in an autosomal recessive mode of inheritance with the patient being homozygous and both unaffected parents being heterozygous. The *ALPK3* variant observed in our study has not previously been reported in a homozygous state in the Database for Short Genetic Variartions (dbSNP) (Sherry et al. 2001), Exome Variant Server, National Heart, Lung, and Blood Institute (NHLBI) GO Exome Sequencing Project (ESP), Seattle, WA, ExAC (Lek et al. 2016), or 1000 Genomes (1000 Genomes Project Consortium et al. 2015) databases nor has it been observed within a cohort of 3000 subjects with nonneurological diseases who were whole-exome-sequenced at Yale School of Medicine. However, it has been reported in a heterozygous state twice with 1.755 × 10^−05^ allele frequency in the ExAc database and five times with 2.096 × 10^−5^ allele frequency in the gnomAD browser (Lek et al. 2016). In addition, copy-number variation analysis (based on exome sequencing of the index case) demonstrated no disease-causing large-scale amplifications, deletions, or loss of heterozygosity within the coding regions of the entire genome. Applying American College of Medical Genetics and Genomics and the Association for Molecular Pathology criteria PVS1, PM3, and PM2, we classified the variant as pathogenic (Richards et al. 2015). These findings provide strong genetic evidence that the identified *ALPK3* variant is the disease-causing variant in this family.

**Table 1.** Variants detected via whole-exome sequencing

Gene symbol	Chromosome: position start	Reference DNA	Alternative DNA	HGVSc	HGVSp	Variant type	Existing variation	Genotype (heterozygous/homozygous)
*ALPK3*	15:85,383,921	AC	A	c.2018delC	p.Gln675SerfsX30	Frameshift		Homozygous
*ARHGAP6*	X:11,157,357	C	T	c.2551G>A	p.Glu851Lys	Missense		Homozygous
*DOCK11*	X:117,702,043	A	G	c.953A>G	p.Tyr318Cys	Missense		Homozygous
*HEPH*	X:65,417,725	G	C	c.1573G>C	p.Asp525His	Missense	rs3747359	Homozygous
*KANSL1L*	2:210,887,679	A	AT	c.2831dupA	p.Asn944LysfsX2	Frameshift		Homozygous
*POU3F4*	X:82,763,774	G	C	c.442G>C	p.Gly148Arg	Missense	Two hemizygotes with 0.00006970 allele frequency and four heterozygotic states in ExAC database	Homozygous
*RRAGB*	X:55,784,736	A	G	c.1001A>G	p.Glu334Gly	Missense		Homozygous
*TNK2*	3:195,594,921	C	T	c.2299G>A	p.Asp767Asn	Missense	rs373598238	Homozygous

HGVSc, Human Genome Variation Society coding sequence name; HGVSp, Human Genome Variation Society protein sequence name; ExAC, Exome Aggregation Consortium.

## DISCUSSION

Human α-protein kinase 3 (*ALPK3*) consists of 14 exons and encodes a 1907-aa protein that contains one α-type protein kinase domain and two Ig-like (immunoglobulin-like) domains (Fig. 1D). Although the specific role of *ALPK3* remains unclear, it may act as a regulator of cardiac transcription factors such as HEY2 (Hosoda et al. 2001; Almomani et al. 2016). Notably, Van Sligtenhorst et al. (2012) observed cardiomyopathy in mice deficient for *Alpk3* that were otherwise phenotypically normal. *Alpk3*^−/−^ mice exhibited nonprogressive cardiomyopathy that had features of both hypertrophic and dilated forms, which is consistent with observations in the patient of our study.

Although hypertrophic and dilated cardiomyopathies were classically described as distinct entities, it has been suggested that these two types of cardiomyopathy may represent different stages within a pathological spectrum (Freeman et al. 2001). Our patient presented with congenital cardiomyopathy that was diagnosed during fetal life similar with previous reports. However, following birth of the patient, cardiac morphology, which was found to be dilated during the fetal life, progressed to HCM. Although it has been previously shown that cases with HCM may progress to DCM (Spirito et al. 1987; Hecht et al. 1993), we did not find any report in the literature showing progression of DCM to HCM. Almomani et al. (2016) also reported that the patients who were lost during the intrauterine life or immediately following the delivery (three out of five patients) exhibited features of DCM or a combination of DCM and HCM. Interestingly, two individuals that were heterozygous carriers for the reported *ALPK3* variant (c.5295G.A; p.Trp1765*) from the Almomani report were diagnosed with an atypical form of HCM during early adulthood. In our study, cardiac examination of heterozygous carriers revealed no evidence of cardiomyopathy, however as suggested by Almomani et al., *ALPK3* pathogenic variant carriers might have an increased risk of developing cardiomyopathy. Clarification of the risk status of heterozygous carriers with *ALPK3* mutations will require further genotype/phenotype correlations with larger cohorts. Of note, Phelan et al., in their report, extended the *ALPK3* phenotype to include congenital pterygia which is not seen in the presented case (Table 2).

**Table 2.** Summary of clinical and molecular findings in *ALPK3* deficiency

Previous paper	Almomani et al. (2016)	Phelan et al. (2016)	Present report (2017)	
Number of families	3	1	1	
Number of cases	5	2	2	
Ethnic origin	Dutch, Moroccan, Turkish	Pakistani	Turkish	
Parental consanguinity	Consanguineous	Consanguineous (first cousins)	Same village	
Sex	Female (3), male (2)	Female, male	Male	Male
Age of diagnosis	At 20 wk of gestation to 4 years of age	Early infancy	Intrauterine	21 wk of gestation
Predominant CMP type	DCM or mixed DCM to HCM phenotypes	Initially left ventricular dilation and then syndromic HCM	NA	Initially DCM and then HCM phenotype
Survival	Intrauterine fetal death at 35 wk to died at 5 days (3/5), alive (ages >7 years, 11 years) (2/5)	Alive (age >18, ?) (2/2)	Died at 30 wk of gestation	Alive (age >3 years)
Other findings	Massive skin edema	Multiple pterygia with skeletal muscle underdevelopment	None	Dysmorphic face
Carrier phenotype/genotype/CMP type/mean age	Affected (2/10)/c.5294G>A, p.Trp1765*/HCM/44 years old	Normal (4/4)	Normal (2/2)	
Genotype	c.4736-1G>A, p.Val1579Glyfs*30; c.3781C>T, p.Arg1261*; c.5294G>A, p.Trp1765*	c.3792G.A, p.Trp1264*	Not tested	c.2018delC, p.Gln675Serfs*30

NA, not applicable; DCM, dilated cardiomyopathy; HCM, hypertrophic cardiomyopathy.

Previous studies have investigated the downstream molecular mechanisms of cardiomyopathy-associated gene mutations. Induced pluripotent stem cell–derived cardiomyocytes (iPSC-CMs) were used to successfully recapitulate the disease phenotype from genes associated with DCM (Sylvius and Tesson 2006; Siu et al. 2012; Sun et al. 2012; Tse et al. 2013) and HCM (Lan et al. 2013). Phelan et al. showed that *ALPK3* knockout human embryonic stem cells (hESCs) recapitulate the cellular phenotype of patient-derived iPSCs. Heterozygous correction of *ALPK3* was sufficient to fully restore a wild-type phenotype in the patient-derived cells. Electron microscopy of *ALPK3*-mutant and control cardiomyocytes derived from both iPSC and hESC lines demonstrated sarcomeric disorganization and abnormal intercalated disc morphology in mutant cardiomyocytes.

### Conclusions

We provide further evidence for involvement of *ALPK3* in cardiomyopathy, as well as identify a unique progression of DCM to HCM. This study underscores the essential role of genetic testing in congenital cardiomyopathy, which should guide appropriate counseling and screening of other family members. Because inherited cardiomyopathies are frequently early-onset and are a major contributor to morbidity and mortality in the young, identification of a pathogenic variant can lead to individualized approaches including symptom management and lifestyle recommendations, family screening, and intervention at a preventive stage.

## METHODS

### DNA Extraction

Blood samples were collected from the infant and his parents. DNA was extracted using the commercially available Gentra Puregene Blood Kit from QIAGEN.

### Whole-Exome Capture, Sequencing, and Data Analysis

Exome capture for all samples was performed using the NimbleGen 2.1M human exome array (Roche Nimblegen Inc.) according to the manufacturer's protocol with modifications, described previously (Choi et al. 2009; Bilgüvar et al. 2010). Exome library sequencing was performed using an Illumina HiSeq2000 with barcoding technology, paired-end analysis, and six samples per lane. Image analysis and subsequent base-calling was performed using the Illumina pipeline (version 1.8). Analysis of the sequencing data was performed according to previously described in-house bioinformatics pipelines (Clark et al. 2013). Briefly, sequence reads that met Illumina quality standards were analyzed and aligned to the human genome reference sequence (version GRCh37, as utilized in phase 1 of the 1000 Genomes Project) using a hybrid of Stampy (Lunter and Goodson 2011) and Burrows–Wheeler alignment (BWA) (Li and Durbin 2009). Variant calling of single-nucleotide variants (SNVs) and small indels was accomplished using the Unified Genotyper algorithm from the Genome Analysis Toolkit (GATK) (DePristo et al. 2011). We annotated variant alleles using the Ensembl database (version 66) and Variant Effect Predictor (v2.4) tool (McLaren et al. 2010). To detect HBD segments, we used an algorithm implemented in BEAGLE v3.3.2.(Browning and Browning 2010). We adopted the default settings and ran the algorithm 10 times with different random seeds as suggested by the authors. We filtered HBD segments with final length of <0.25 cM.

### Sanger Sequencing

Coding regions and exon–intron boundaries of *ALPK3* were evaluated by Sanger sequencing using standard protocols (Supplemental Table 2). Amplicons were cycle-sequenced on ABI 9800 Fast Thermo cyclers, and post–cycle sequencing cleanup was carried out with the CleanSEQ System (Beckman Coulter Genomics). The amplicons were analyzed with a 3730×L DNA Analyzer (Applied Biosystems Inc).

### Copy-Number Variation Analysis

The depth of coverage log ratio between patient sample and control samples were calculated using GATK-Depth of Coverage tool. Segments with copy-number variations (CNVs) were identified from the log ratio of depth of coverage using the ExomeCNV R package (Sathirapongsasuti et al. 2011). False-positive CNV events were identified and corrected for by calculating minor allele frequencies in each CNV segment.

## ADDITIONAL INFORMATION

### Data Deposition and Access

Whole-exome sequencing data are not publicly available because consent could not be obtained. The variant associated with the phenotype has been submitted to ClinVar to be deposited and can be found under accession number SCV000540917 (https://www.ncbi.nlm.nih.gov/clinvar/).

### Ethics Statement

The study protocol was approved by the Yale School of Medicine Human Investigation Committee (HIC) (protocol number 0908005592). Institutional review board approvals for genetic studies, along with written consent from all study subjects, were obtained by the referring physicians at the participating institutions. Informed consent to publish clinical data of the patient was signed by the parents.

### Acknowledgments

We thank the reported family for participating in this study.

### Author Contributions

A.O.C. had full access to all data in this study and takes responsibility for the integrity of the data and the accuracy of the data analysis. M.G., A.O.C., and K.B. contributed to the study concept and design and the overall acquisition, analysis, or interpretation of data. R*.*G.S., T.Y., A.B., H.K., and Y.Y. contributed to patient recruitment. A.O.C. contributed to WES data analysis, and interpretation. K.Y. contributed to the in-house WES pipeline. A.S.H. contributed to the CNV analysis. A.O.C. and E.U. contributed to the Sanger sequencing. A.O.C., K.B., J.F.B., and M.W.Y. contributed to the drafting of the manuscript. All authors contributed to the critical revision of the manuscript for important intellectual content. M.G. obtained funding and contributed to study supervision.

### Funding

This work was supported by the Yale Program on Neurogenetics and the Yale Center for Mendelian Disorders (U54HG006504), the National Institutes of Health (NIH) Medical Scientist Training Program Grant T32GM007205, and the Gregory M. Kiez and Mehmet Kutman Foundation (M.G.).

### Competing Interest Statement

The authors have declared no competing interest.

### Referees

Dean G. Phelan

Alireza Haghighi

Anonymous

## Supplementary Material

Supplemental Material
