# Supplementary material for: ALPK3 gene mutation in a patient with congenital cardiomyopathy and dysmorphic features
Source: Cold Spring Harb Mol Case Stud. 2017 Sep;3(5):a001859. doi: 10.1101/mcs.a001859 (PMC5593152; doi:10.1101/mcs.a001859)
Supplement: Supplemental Material [file supp_3_5_a001859__index.html]

ALPK3 gene mutation in a patient with congenital cardiomyopathy and dysmorphic features — Supplemental Material 

# *ALPK3* gene mutation in a patient with congenital cardiomyopathy and dysmorphic features

## Supplemental Material

- Supplemental\_Tables.docx
