# Supplementary material for: ALPK3 gene mutation in a patient with congenital cardiomyopathy and dysmorphic features
Source: Cold Spring Harb Mol Case Stud. 2017 Sep;3(5):a001859. doi: 10.1101/mcs.a001859 (PMC5593152; doi:10.1101/mcs.a001859)
Supplement: Supplemental Material [file supp_mcs.a001859_Supplemental_Tables.docx]

| **Number of lanes** | | 1 |
| --- | --- | --- |
| **Read Type (SR/PE)** | | PE |
| **Read length** | | 74 |
| **Total number of reads (millions)** | | 103.361.136 |
| **Exome Qualitymetrics** | **% mapped to the exome** | 54,41% |
|  | **Mean Target Coverage** | 100,61 |
|  | **% of bases covered 0X** | 0,52% |
|  | **% of bases covered at least 20X** | 89% |
|  | **Mismatches (%)** | 0,22% |
|  | **Duplicates (%)** | 4,02% |

**Supplementary Table 1.** Exome qualitymetrics of the index patient

**Supplementary Table 2**. Primers are used for segregation analysis and screening

| ALPK3_E1_1F | AATTGAGCCCCTAATCTATGC | ALPK3_E1_1R | GTCACCTGCTGCTGTCCC |
| --- | --- | --- | --- |
| ALPK3_E1_2F | CGCTACTGCAGACACCAGG | ALPK3_E1_2R | CCCTCACAGGGACTGATCC |
| ALPK3_E2_F | GTTTGCGACTGTTGATTTCG | ALPK3_E2_R | CTCAATGCCCAGACCCAAG |
| ALPK3_E3_F | AGGCTGTGCTGTTTGTTGTG | ALPK3_E3_R | ATTCCTGTCCTCACAGCCTC |
| ALPK3_E4_F | TTCCCATTATTTTGGAACTGG | ALPK3_E4_R | GACTGCTCCATTTCTGAGGG |
| ALPK3_E5_1F | CTCTGAACGGCTCTGGCTG | ALPK3_E5_1R | GGCGTCACAGATGTATGTCAG |
| ALPK3_E5_2F | CTGCTCAGCACTCAGGTTTG | ALPK3_E5_2R | ACTCAGGGTCTGGGGTCC |
| ALPK3_E5_3F | TGTGGGCACTCCAGACAAG | ALPK3_E5_3R | GCAGTTGCAGCAAGAGTTTC |
| ALPK3_E6_1F | GGATGCCAGACTGGAAAGAG | ALPK3_E6_1R | TTCAGAGGAGCTCTAGGACCC |
| ALPK3_E6_2F | AGAGAAGGGGACGCAGTCAG | ALPK3_E6_2R | GTGGCCATGGTTTCTGGTG |
| ALPK3_E6_3F | CGACTTCTCAGCACGGGAG | ALPK3_E6_3R | ACCAGCCATGCTGCTCTC |
| ALPK3_E6_4F | TGTGGTAGACGAGGAGGACC | ALPK3_E6_4R | CAGCAGTGTGCTTTCTGTGG |
| ALPK3_E7_F | CTTTTCCACCAAGGACACCC | ALPK3_E7_R | CGGTATTACTGCAATCAGGG |
| ALPK3_E8-9_F | GCTTAGGACCACAGTCTGCC | ALPK3_E8-9_R | CAAGCCCTTAAAGAGGGTCC |
| ALPK3_E10_F | CCACATTGGTGAGACAGGAG | ALPK3_E10_R | CTAACAGGACCTGGGCTGG |
| ALPK3_E11_F | GGTAGCCCACTCACTTAGGG | ALPK3_E11_R | CCTGGGAGGCTGTGAAATC |
